# Electrospun Poly(caprolactone)/Poly(ethylene oxide) Membranes Incorporating Green‐Synthesized Zinc Oxide Nanoparticles for Enhanced Wound Healing Applications

**DOI:** 10.1002/smsc.202500562

**Published:** 2026-06-09

**Authors:** Ronaldo Silveira, Henrique Borba Modolon, Edgar Andrés Chavarriaga, Alex Arbey Lopera, Jaqueline Leite Vieira, Milena Botelho Pereira Soares, Josiane Dantas Viana Barbosa, Elidio Angioletto, Tiago Bender Wermuth, Oscar Rubem Klegues Montedo, Sabrina Arcaro

**Affiliations:** ^1^ Laboratório de Cerâmica Técnica (CerTec) Grupo de Pesquisa em Biomateriais e Materiais Nanoestruturados Universidade do Extremo Sul Catarinense (UNESC) Criciúma Brazil; ^2^ Programa de Pós‐Graduação em Ciência e Engenharia de Materiais (PPGCEM) Universidade do Extremo Sul Catarinense (UNESC) Criciúma Brazil; ^3^ Departamento de Fundamentación Básica Institución Universitaria Pascual Bravo Medellín Colombia; ^4^ Laboratorio de Materiales Cerámicos y Vítreos Departamento de Física Universidad Nacional de Colombia – Sede Medellín Medellín Colombia; ^5^ Gonçalo Moniz Institute Oswaldo Cruz Foundation (FIOCRUZ) Salvador Brazil; ^6^ SENAI Institute for Innovation in Advanced Health Systems (ISI SAS) SENAI CIMATEC University Center Salvador Brazil; ^7^ Laboratório de Desenvolvimento de Materiais Antimicrobianos – (LADEBIMA) Universidade do Extremo Sul Catarinense (UNESC) Criciúma Brazil

**Keywords:** antibacterial activity, electrospinning, green synthesis, wound healing, zinc oxide nanoparticles

## Abstract

Infections are the most frequent complication that impair normal skin repair, often leading to chronic wounds. To overcome this challenge, zinc oxide nanoparticles (ZnO NPs) were synthesized via green combustion using *Moringa oleifera* extract as fuel. These nanoparticles were incorporated into a poly(caprolactone)/poly(ethylene oxide) (PCL/PEO) membrane fabricated via electrospinning. The composite membranes were characterized by water contact angle, fluid absorption, morphology (SEM), cytotoxicity, cell proliferation (scratch assay), and antibacterial activity against *Staphylococcus aureus* and *Escherichia coli*. The PCL/PEO membrane containing 5% ZnO exhibited optimal characteristics, including hydrophilicity, water uptake capacity exceeding 300%, and a morphology that is suitable for wound healing applications, with an average fiber diameter of 2.93 ± 2.05 µm, pore size of 21.46 ± 8.19 µm, and porosity exceeding 75%. Additionally, the membrane demonstrated excellent biocompatibility with murine fibroblasts, achieving a cell viability of 100%, and exhibited antibacterial activity against *Staphylococcus aureus* and *Escherichia coli*, with inhibition zones of 2.0 and 1.0 mm, respectively. The green synthesized moringa‐ZnO imparted outstanding properties to the electrospun membrane, making the system PCL/PEO/moringa‐ZnO a promising candidate for advanced wound dressings capable of promoting accelerated wound closure and treating infected wounds.

## Graphical abstract

Green‐synthesized zinc oxide nanoparticles obtained via green‐combustion synthesis with *Moringa oleifera* leaves extract are incorporated into electrospun poly(caprolactone)/poly(ethylene oxide) (PCL/PEO) membranes for wound healing. The resulting composite exhibits enhanced hydrophilicity, high fluid absorption, excellent biocompatibility, and antibacterial activity against *Staphylococcus aureus* and *Escherichia* *coli*, highlighting its potential as an advanced dressing for infected and chronic wounds.

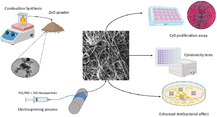

## 1 Introduction

The skin acts as a primary protective barrier but is vulnerable to injuries ranging from minor lesions to severe wounds. Although wound healing is generally a well‐regulated biological process, factors such as infection, inflammation, and systemic conditions, including diabetes and malnutrition, can impair tissue repair and lead to chronic wounds [1, 2, 3]. Infection represents the most common complication during skin regeneration. While the presence of bacteria in wounds is expected, bacterial loads exceeding 10^5^ cells per gram of tissue can impair healing and promote chronic wound formation. These wounds are characterized by a prolonged inflammatory phase, which hinders progression to subsequent healing stages [4, 5]. Antibiotic‐based therapies remain the standard treatment; however, their widespread use raises concerns regarding antimicrobial resistance and adverse effects [6, 7]. Additionally, conventional wound dressings, such as gauzes and bandages, provide limited exudate management and often require frequent replacement, which may compromise patient comfort and healing efficiency [8, 9].

Effective wound dressings play an important role in accelerating the healing process, particularly for chronic wounds. An ideal functional dressing should be biocompatible, mechanically strength to protect the wound, and exhibit cytotoxicity while promoting high cell adhesion. Additionally, it should facilitate gas exchange with the environment, prevent bacteria infiltration into the wound site, and possess hydrophilicity to maintain adequate moisture levels and manage exudates. These combined characteristics create an optimal environment for tissue regeneration, supporting efficient wound healing [10, 11]. Electrospinning is a promising technique for producing fibrous membranes with precise morphological control, including high porosity, tunable fiber diameters, and adjustable pore sizes. This ability to tailor membrane's morphology allows the production of structures that mimic the extracellular matrix (ECM), which have demonstrated superior regenerative capabilities compared to conventional dressings [3]. Maintaining fiber diameters ranging from 300 to 1000 nm, controlled porosity between 60% and 90%, and pore sizes of 20–125 µm, these fibrous membranes promote cell adhesion, enhance tissue regeneration and are ideal for skin repair [12, 13, 14].

Bioabsorbable and biocompatible polymers are widely used in the development of advanced wound dressings. Poly(caprolactone) (PCL) is a commonly employed polymer due to its excellent biocompatibility, mechanical properties, and slow biodegradability. However, its high hydrophobicity limits its use in pure form for skin regeneration applications [1]. To overcome this limitation, PCL is often combined with other biocompatible polymers such as chitosan [15], cellulose acetate [16], alginate [17], polyvinyl alcohol (PVA) [18], and poly(ethylene oxide) (PEO) [19, 20, 21]. These composites enhance the material's wettability and overall performance, making them suitable for wound healing applications.

Zinc oxide nanoparticles (ZnO NPs) have emerged as a promising therapeutic agent for skin wound healing. ZnO NPs exhibit excellent antibacterial, anti‐inflammatory, and wound‐healing properties, making them highly effective in treating skin injuries [22, 23, 24]. Unlike silver nanoparticles (Ag NPs), which can aggregate and exhibit higher toxicity to mammalian cells [1, 25, 26, 27], ZnO NPs approved by FDA and are considered safe for dermal applications due to their low toxicity [28, 29]. Zinc is an essential trace element for skin health, and its therapeutic potential has been widely recognized in pharmaceutical and cosmetic products, including sunscreens, healing ointments, and acne treatments [30, 31]. The incorporation of ZnO NPs into wound dressings offers a viable alternative to antibiotics, addressing concerns related to antibiotic resistance and providing a safer, more effective solution for wound care [32, 33].

Green synthesis has emerged as a promising and sustainable approach to produce nanoparticles, offering significant advantages such as cost‐effectiveness, environmental friendliness, and enhanced particle stabilization. Among the various natural sources explored for green synthesis, *Moringa oleifera* stands out due to its rich composition of bioactive compounds, including carotenoids, alkaloids, polyphenols, and flavonoids. These compounds not only act as reducing and stabilizing agents during nanoparticle synthesis but also impart additional functional properties, such as antibacterial, antioxidant, and anti‐inflammatory effects [34, 35]. *Moringa oleifera* has demonstrated remarkable potential in diverse applications, including anti‐cancer therapies, antimicrobial treatments, and wound healing [36, 37]. Specifically, the use of *Moringa oleifera* extract in the combustion synthesis of ZnO NPs represents a novel and highly promising approach [37].

Thus, this study aims to synthesize ZnO NPs using a green combustion method with *Moringa oleifera* extract and incorporate them into electrospun membranes for wound healing applications, especially for infected wounds. The resulting nanocomposite membranes combine the antibacterial and anti‐inflammatory properties of ZnO NPs with the structural and functional advantages of electrospun fibers of PCL/PEO, such as high porosity, good wettability, gas exchange, and ECM‐like morphology that enhances cell proliferation. Although there are studies in the literature describing the production of membranes of PCL with ZnO NPs, no research has been reported on constructing a membrane with the combination of PCL/PEO with ZnO NPs obtained by combustion solution synthesis using *Moringa oleifera* extract. This unique composition has not been previously explored, presenting an opportunity to develop a novel biomaterial with potentially advantageous properties for accelerated wound healing of infected wounds, providing a potential alternative to conventional antibiotic‐based treatments.

## 2 Materials and Methods

ZnO NPs were synthesized by solution combustion method. These nanoparticles were characterized and further utilized as active content in membranes produced by the electrospinning technique, using PCL and PEO. Then, evaluations of wettability, morphology and mechanical tests, cytotoxicity tests, cell proliferation and antimicrobial activity tests were carried out.

### 2.1 Zinc Oxide Synthesis

The combustion synthesis procedure is reported in our previous work [38]. In brief, for the preparation of the ZnO NPs, *Moringa oleifera* extract was used as a natural oxidant in the combustion solution synthesis. For the extraction 20 g of dry leaves, purchased from a local market in Medellín, Colombia, were combined with 100 mL of deionized water as a solvent. The mixture undergoes an ultrasonic treatment for 30 min to ensure the maximum extraction efficiency using an ultrasonic processor (sonics materials VC‐750‐220, 750 W and 20 kHz), allowing the water to penetrate the plant material and extract a diverse range of compounds. After the treatment the dry leaves were separated by simple filtration, resulting in a clarified extract of moringa leaves.

In the synthesis process 4.3 g of ammonium nitrate was utilized as an extra oxidant in addition to the leaf extract; 7.442 g of Zn(NO_3_)_2_ · 6H_2_O was utilized as de zinc source. The mixture was heated for water evaporation until the ignition point for the solution combustion.

### 2.2 Zinc Oxide Characterization

For the characterization of the obtained powders, X‐ray diffraction (XRD) analysis was employed. For this analysis the X‐ray diffractometer (Model D8 Bruker, Germany; Cu Kα radiation) was utilized. The crystallographic phases were analyzed in comparison with standard reference patterns to ensure the ZnO purity. Fourier transform infrared spectroscopy (FTIR) with attenuated total reflectance (ATR‐FTIR, Tensor II model, Bruker, Germany) was utilized to identify the functional groups, the spectroscopy was conducted at wavelengths ranging from 4000 to 400 cm^−1^. For the measurement of the surface area of the ZnO powders the Quantachrome NOVA 2200E BET Surface Area Analyzer (USA), utilizing nitrogen absorption. The morphology of ZnO powders was studied using a scanning electron microscope (SEM, JEOL JSM‐5900, Japan), which operated at an acceleration voltage of 15 kV and a working distance (WD) of 10 nm. Transmission electron microscopy (TEM, Tecnai G2‐20 – FEI SuperTwin, The Netherlands) was also used, operated at 200 kV, and the images were analyzed with Image‐J software.

Crystallite size was estimated using Sherrer equation (Equation (1)), based on (110) plane of the obtained XRD pattern [39]. The lattice parameters *a* and *c* were determined using Bragg's diffraction equation (Equation (2)) and the interplanar spacing equation (Equation (3)) for the hexagonal system

(1) $$D=\frac{K\lambda }{\beta \cos\theta }$$

where *D* is the crystallite size, *β* is the full width at half maxima, *θ* is the Bragg angle, *λ* is the wavelength of Cu‐Kα radiation, and *K* is the Scherrer´s constant (Shape factor), which can be estimated as 0.94.

(2) $$d_{hkl}=\frac{n\lambda }{2\sin\theta }$$

(3) $$\frac{1}{d_{hkl}}=\frac{4(h^{2}+k^{2}+l^{2})}{3a^{2}}+\frac{l^{2}}{c^{2}}$$

where $d_{hkl}$ is the interplanar distance, θ is the incidence angle of X‐rays, n is the order of diffraction, λ is the wavelength of the X‐ray beans, and *h*, *k*, and *l* are the Miller indices.

### 2.3 Membrane Preparation

PCL, molecular weight = 80,000 g/mol (Sigma Aldrich, USA) and PEO, molecular weight = 900,000 g/mol (Sigma Aldrich, USA) were used as polymers for membrane production. Chloroform 99.80% purity (LabSynth, Brazil) was used as solvent.

For preparing the membranes with different amounts of ZnO addition (0, 1, 2.5, and 5 wt% relative to the polymer mass), the nanoparticle powder was dissolved in chloroform, dispersed for 5 min with sonication (Desruptor, Ultronique). After that, the solution formed by chloroform and ZnO NPs was used as a solvent for dissolving the polymers. A mixture of PCL and PEO (7:1, w/w) was used, resulting in a polymeric solution of 8% (w/v). The solution was maintained under continuous stirring for 18 h until the complete dissolution of the polymers. A pure PCL membrane was also prepared for comparison, with 8% polymer content and pure chloroform as solvent. The membrane without ZnO addition was labeled as PCL/PEOZ0, and membranes with 1, 2.5, and 5 wt% of ZnO content were respectively labeled as PCL/PEOZ1, PCL/PEOZ2.5 and PCL/PEOZ5.

The electrospinning process was conducted in a DBM *Eletrotech Eletrospinner* device (Brazil), with horizontal layout and drum collector with rotation speed control. The polymeric solutions were placed into a 10 mL syringe, delivered to the tip with an automatic pump. A metallic needle with blunt tip (25 mm × 0.7 mm) was used. The solutions were electrospun with a controlled flow rate of 0.07 mL/min, tip to collector distance of 15 cm, 20 kV tension, 280 rpm of collector rotation speed. All samples were prepared under controlled environmental conditions, at a temperature of 21°C and relative air moisture ranging from 55% to 70%.

### 2.4 *Fourier Transform Infrared* (*FTIR*)

For structural analysis of the membranes of PCL, PCL/PEO, and PCL/PEO with ZnO from 1 to 5 wt% the FTIR was assessed using a Bruker Tensor II spectrophotometer (Germany) with an ATR‐FTIR accessory. The spectra were collected with a scanning range of 4000–400 cm^−1^.

### 2.5 Morphology

Morphology of the membranes was characterized in terms of fiber diameter, pore size, and porosity. Membrane images were obtained by SEM (Zeiss EVO MA 10, Germany) operated at 8 kV. The linear intercept method was utilized to estimate the pore size and fiber diameter, utilizing the obtained images and Image‐J software. Measurements were performed on 300 fibers and 100 pores sampled from different membrane regions. Equation (4), which relates the apparent density and true density, was used o calculate the membrane porosity. Apparent density ($D_{\mathrm{AP}}$) was determined from the mass of membrane sections, with dimensions measured with a digital caliper (Mitutoyo, Japan). True density, $D_{\mathrm{True}}$, was determined from each sample using helium gas pycnometry (AccuPyc 1340, Micromeritics, USA).

(4) $$P=\left(1-\;\frac{D_{\mathrm{AP}}}{D_{\mathrm{True}}}\right)\times 100$$

### 2.6 Wettability and Water Uptake

The water contact angle method was used to analyze the wettability of the membranes. A high‐resolution microcamera was used for image capture. A micropipette was employed, delivering a 10 µL droplet of distilled water onto each membrane surface. The angles were measured using the Image‐J software, at the exact contact of the droplet and after 5 s.

To evaluate material's capacity of absorbing exudates from the wound site, the water uptake was assessed using Equation (5), where $W_{0}$ (g) is the mass of the dry membranes and $W_{1}$ (g) is the mass of the wet membrane after 24 h of immersion in distilled water at 37°C. The test was conducted in triplicate and the results are expressed as percentage of water absorbed in relation to the original mass.

(5) $$\text{Water Uptake }\left(\%\right)=\;\frac{W_{1}-W_{0}}{W_{0}}$$

### 2.7 In Vitro Cytotoxicity of Membranes

Immortalized murine fibroblasts (NiH3T3) were utilized for cell viability experiments and the MTT assay described by Mosmann (1983) was employed. The cells were cultured in 25 cm^2^ polymer flasks, maintained in Dulbecco's Modified Eagle Medium (DMEM; Life Technologies, GIBCO, Thermo Fisher Scientific) supplemented with 10% fetal bovine serum (FBS; GIBCO, Thermo Fisher Scientific) and 1% penicillin/streptomycin (Thermo Fisher Scientific). Cells were incubated at 37°C with a 5% CO_2_ atmosphere. Once the desired confluence was reached, the DMEM was removed, and 4 mL of trypsin was added to detach the cells. Trypsin was neutralized with 4 mL of DMEM, and cell count was performed using a Neubauer chamber.

In a 96‐well plate, 100 μL of cell suspension, diluted in DMEM to a final concentration of 1 × 10^4^ cells/well, was added to each well. Incubation was carried out in a humidified incubator at 37°C with 5% CO_2_ atmosphere. Afterward, the membrane samples were incubated for 24 h under the same conditions as mentioned above.

A MTT solution was prepared at 0.5 mg/mL in phosphate buffered solution (PBS; Sigma Aldrich, Missouri, EUA), and 100 µL of this solution was added to each well after the membranes were removed. Following a 3 h incubation under the same conditions, the MTT was removed, and 100 µL of isopropyl alcohol was added to solubilize the formazan crystals. Absorbance was measured in a spectrophotometer at a 570 nm wavelength. Experiments were conducted in triplicate and the results were expressed in percentage of cell viability.

### 2.8 In Vitro Cell Migration Assay

To evaluate cell migration, murine fibroblast L929 cells were cultured in a 24‐well plate at a density of 2.5 x 10^5^ cells/well and incubated for 72 h under standard culture conditions (37°C, 5% CO_2_ humidified incubator). A linear scratch was introduced into the confluent monolayer using a sterile pipette tip. After scratching, phosphate buffer saline was utilized to wash and eliminate cellular debris. Each well was subsequently treated with the respective membrane sample, while control wells received no treatment. Images were captured at 0 and 24 h using a digital camera attached to an inverted microscope. The unfilled area was quantified at each time point, with w(i) representing the initial scratched area and *w*(*f*) the remaining area after 24 h, using ImageJ software. The experiment was performed in quadruplicate and the percentage of wound closure was calculated according to Equation (6)

(6) $$\text{Wound Closure }\left(\%\right)=\;\frac{w\left(i\right)-w\left(f\right)}{w\left(i\right)}\times \;100$$

### 2.9 In Vitro Antibacterial Activity of Composite Membranes

Inhibition zone method was utilized for antibacterial activity measurement against Gram‐positive bacteria *Staphylococcus aureus* (ATCC 25923) and Gram‐negative bacteria *Escherichia coli* (ATCC 25922). The microorganism was prepared by streaking into a solid culture medium and inoculation in *brain‐heart‐infusion* (BHI) medium of an isolated colony forming unity (CFU) identified as pure. Incubation conditions were 24 h period at 37°C. In plates containing Plate counting agar, 100 μL of bacterial solution at 1 x 10^6^ CFU/mL was inoculated and spread. Membrane samples were cut into 10 mm × 10 mm pieces, sterilized by ultraviolet light (UV) for 1 h, 30 min each side and then added onto agar surface. Measurements of the inhibition halo formed were taken in triplicate with a digital caliper (Mitutoyo, Japan).

### 2.10 Statistical Analysis

Statistical analyses of the water uptake, cytotoxicity and wound closure data were performed using STATISTICA 12.0 software. One‐way analysis of variance (ANOVA) followed by Tukey's post hoc multiple comparison test was applied. A confidence level of 95% and a significance threshold of *p* < 0.05 were adopted for all analyses, except for the wound closure assay, for which a confidence level of 70% and *p* < 0.30 were considered statistically significant.

## 3 Results and Discussion

### 3.1 Zinc Oxide Characterization

**FIGURE 1:**
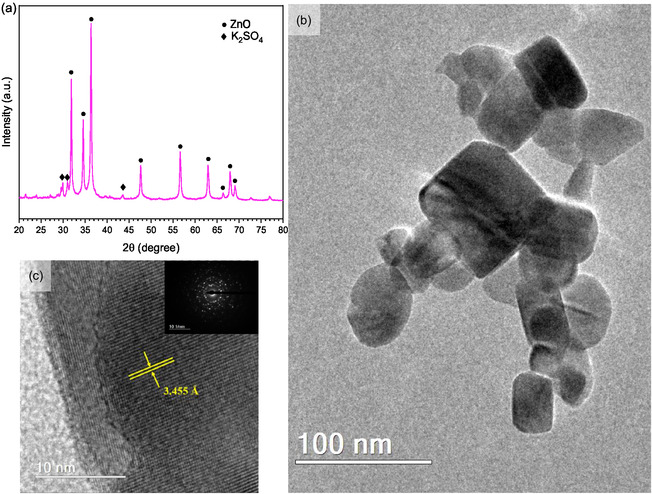
(a) XRD spectra for the ZnO powder. (b) TEM images showing ZnO nanoparticles, scale bar 100 nm. (c) TEM image showing the measured interplanar distance, scale bar 10 nm.

Figure 1a shows the XRD spectrum for the sample obtained by solution combustion synthesis with *Moringa oleifera* extract to confirm the formation of the desired phases.

The sample presents narrow peaks, indicating a powder with high crystallinity. Peaks were observed at 2*θ* angles of approximately 31.96° (100), 34.55° (002), 36.25° (101), 47.69° (102), 56.68° (110), 62.85° (103), 66.55° (200), 68.04° (112), and 69.03° (201), which can be attributed to the hexagonal wurtzite structure with the *P63mc* space group (JCPDS 36‐1451). Additionally, the presence of a secondary crystalline phase was identified by diffraction peaks at 2θ values of approximately 29.96°, 30.99°, and 43.53°, which can be assigned to arcanite (K_2_SO_4_) (JCPDS no. 00‐005‐0613). The occurrence of this phase is attributed to the use of *Moringa* leaf extract in the combustion synthesis, which can introduce trace inorganic species into the nanoparticle structure [38]. Similar incorporation of secondary mineral phases in nanoparticles synthesized via green combustion routes has been reported by Sheibani et al. [40], which reported the presence of potassium chloride (KCl) in the ZnO nanoparticles, identified in the XRD analysis, as a residue from the combustion synthesis using *Allium schoenoprasum* leave extract. Crystallite size was estimated at 33.1 nm. The calculated values of *a* = 3.2397 Å and *c* = 5.1836 Å are consistent with previous reports, confirming the formation of the desired phase.

BET analysis indicates a ZnO powder with a surface area of 49.944 m^2^/g. The high surface area can be attributed to the gas formation from the decomposition of ammonium nitrate that is utilized as an extra oxidizer in the combustion reaction, which is often linked to the formation of porous nanoparticles with low aggregation and high surface areas [41]. Figure 1b shows TEM images of the ZnO powder. Crystallite size was measured as 32.25 ± 12.22 nm, utilizing ImageJ software and taking 50 particles for the measurement. The distance between crystallographic planes taken from the TEM image in Figure 1c was found to be 3.455 Å. These results align with the crystallite size estimated by Sherrer equation and the estimated lattice parameter *a* from the (100) crystallographic plane, obtained from the DRX.

### 3.2 Fourier Transform Infrared (FTIR)

**FIGURE 2:**
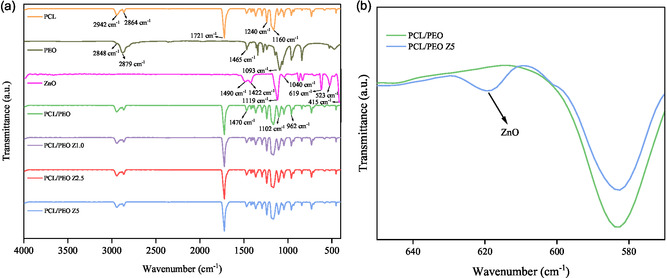
(a) FTIR spectra of PCL, PEO, ZnO, and the blends PCL/PEO, PCL/PEOZ1.0, PCL/PEOZ2.5, and PCL/PEOZ5. (b) FTIR spectra of the PCL/PEO and PCL/PEOZ5 demonstrating that ZnO NPs were implemented on the membrane matrix.

FTIR spectra of PCL, PEO, ZnO NPs, and PCL/PEO electrospun membranes with ZnO at different concentrations (1, 2.5, and 5 wt%) are presented in Figure 2a.

For the PCL, its specific bands were observed at 1160 and 1240 cm^−1^, which corresponds to the symmetric and asymmetric stretching vibrations of the ester linkages C–O, the strong peak observed at 1721 cm^−1^ corresponds to the stretching vibrations of the ester carbonyl groups, the bands at 2942 and 2864 cm^−1^ corresponds to the symmetric and asymmetric stretching of C–H [42].

The characteristics bands for the PEO can be observed in a strong broad band at 1093 cm^−1^ which corresponds to the asymmetric stretching vibrations of C–O–C bonds, the band observed at 1465 cm^−1^ can be associated with the scissoring vibration of CH_2_, the bands observed at 2848 and 2879 cm^−1^, corresponds to the symmetric and asymmetric stretching vibrations also associated with CH_2_ conformation groups [42, 43].

The FTIR spectrum of the ZnO NP sample exhibited characteristic vibrational bands at 415, 523, and 619 cm^−1^, corresponding to the Zn–O stretching modes. These assignments are consistent with previous reports on ZnO NPs, where lattice vibrations are typically reported within this spectral range [44, 45]. Additional absorption bands were detected, indicating the presence of secondary functional groups. The presence of carbonate‐related functional groups was identified by bands at 1040 cm^−1^ and 1119 cm^−1^, which correspond to C–O stretching vibrations, likely originating from residual byproducts from the combustion synthesis process. Moreover, the bands at 1119 and 619 cm^−1^ may partially overlap with vibrational modes of sulfate ions SO_4_^2−^ from arcanite, whose bands coincide with those from carbonates and ZnO [46]. The bands at the 1422 and 1490 cm^−1^region were attributed to asymmetric vibrations of nitrate (NO_3_^−^) and deformation modes of ammonium ions (NH_4_^+^). The presence of these bands suggests possible adsorption of nitrogen‐containing species onto ZnO NPs, likely due to residual precursor compounds from ZnO synthesis [40, 47]. The broad O‐H stretching band, typically observed around 3400 cm^−1^ was absent, which can be attributed to the nature of the combustion synthesis, where the high temperature flame effectively eliminates adsorbed moisture and hydroxyl groups from polyphenol groups of the leaves extract [48]. Combined FTIR and XRD analyses indicate that ZnO nanoparticles synthesized via the combustion route exhibit chemical characteristics distinct from those produced by precipitation methods using plant extracts. ZnO nanoparticles obtained by precipitation based green synthesis are reported to leverage key plant‐derived compounds such as polyphenols and flavonoids components into the particle, which appear in the FTIR spectra as bands associated with C=C aromatic ring stretch, C–N of aromatic amines, and C=O groups [49, 50]. In contrast, when the combustion route is adopted for the green synthesis of ZnO using plant extracts, these organic components are decomposed at elevated temperatures, promoting the conversion of zinc precursors into ZnO. Consequently, the resulting FTIR spectra predominantly display bands associated with residual carbon‐ and nitrogen‐containing species, without vibrational features related to aromatic organic compounds [40, 48].

The appearance of different bands observed at 1102, 1470, and 1727 cm^−1^ regions on the PCL/PEO membrane indicates that the blend was successfully obtained, and that no chemical reactions occurred, this can be affirmed by the fact that no new chemical bonds were identified in the FTIR spectra [51].

Figure 2a presents an ampliation to show a comparison between the PCL/PEO and PCL/PEOZ5 membrane. It is possible to observe a new band formation on the 620 cm^−1^ region as the ZnO NPs concentration increases, this can be attributed to the fact that ZnO was successfully implemented in the PCL/PEO matrix [44, 45].

### 3.3 Membrane Morphology

The electrospinning technique enables the fabrication of materials with unique morphologies that are highly suitable for wound healing applications. An ideal wound healing material should promote cell proliferation, prevent bacterial infiltration, facilitate gas exchange with the environment, and maintain optimal moisture levels. A morphology that is similar to that of the skin's extracellular matrix is known to enhance cell proliferation and migration, improving membrane performance. For this purpose, a fiber diameter ranging from 300 nm to a few micrometers, porosity above 60% and an appropriate pore size are desired [13, 14].

**FIGURE 3:**
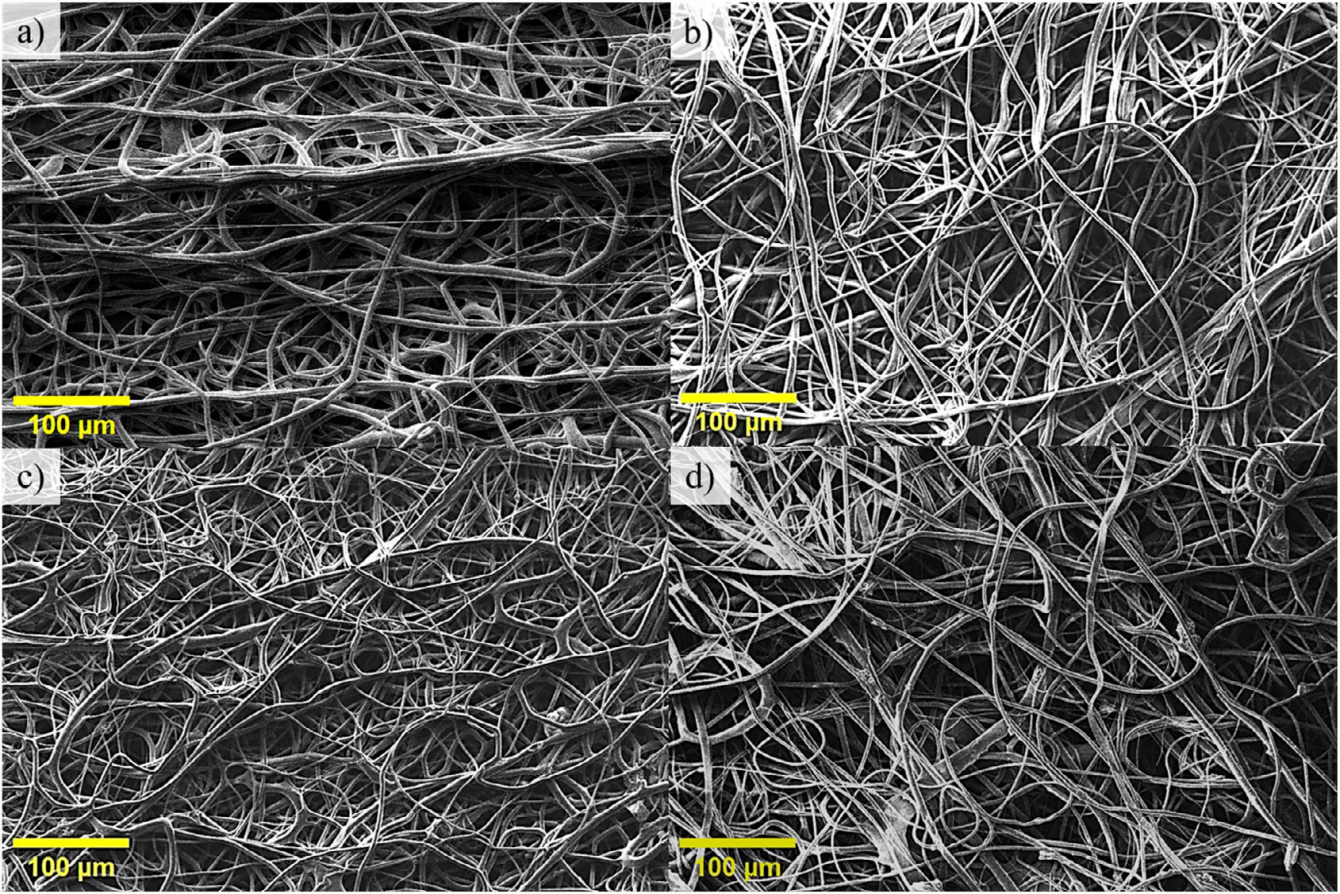
SEM images with 1000x magnification of the obtained membranes with composition: PCL/PEOZ0 (a), PCL/PEOZ1 (b), PCL/PEOZ2.5 (c), and PCL/PEOZ5 (d). Scale bars 100 µm.

The SEM micrographs presented in Figure 3 illustrate the structural characteristics of electrospun membranes with the synthesized ZnO addition in concentrations ranging from 0 to 5 wt% relative to polymer mass.

The fibrous membranes revealed in Figure 3a–d have no visible beads or defects, with smooth and uniform fibers for all the samples. Morphological alterations were verified under ZnO addition, with a slight decrease in fiber diameter in relation to the PCL/PEO membrane with no zinc oxide content. Other morphological properties of the obtained membranes are shown in Table 1.

**TABLE 1:** Morphological properties of the membranes with different compositions.

Sample	Fiber diameter, μm	Porosity, %	Pore size, μm
PCL/PEOZ0	3.20±1.40	73.26±2.52	25.15±9.88
PCL/PEOZ1	2.82±1.76	73.96±1.06	21.27±9.20
PCL/PEOZ2.5	2.92±2.13	77.05±1.35	21.69±9.36
PCL/PEOZ5	2.93±2.05	76.57±0.70	21.46±8.19

Table 1 analysis indicates that all samples have an average fiber diameter in the micrometric scale, PCL/PEOZ0, the membrane without ZnO in the composition possesses the largest fiber diameter, whereas the membranes with ZnO addition have a slightly smaller fiber diameter; however, the standard deviation is higher, indicating a less uniform fiber distribution.

Our study found a reduction in fiber size with ZnO addition that is consistent with Augustine et al. (2014) work, for membranes of PCL with ZnO addition at concentration up to 1 wt% they found a reduction in the mean fiber diameter compared to a membrane of pure PCL. A further addition in the ZnO concentration led to an increase in the average diameter. On the other hand, our study found a reduction in the fiber diameter for membranes with ZnO addition in concentrations up to 5 wt% when compared to membranes without ZnO, with no increase in concentrations above the limit of 1 wt% as in Augustine et al. (2014) work. Fiber reduction upon ZnO addition can be explained by the increase of electrical charges on the fiber surface during the electrospinning process. The increase in surface charges can be compared to the effect of increasing the working voltage, where a higher elongation force is generated from the tip to the collector direction, thus stretching the polymer fibers and causing a diameter reduction. This phenom is also observed in the work of Bozkaya et al. (2022) for an electrospun membrane of PCL and PEO loaded with Ag nanoparticles.

A high porosity can be observed across all the samples, and a slight increase occurred with ZnO addition. The highest pore size is observed on the PCL/PEOZ0 membrane, the addition of ZnO caused a slight reduction in pore size; however, it can be noted that is not related with the concentration. This decrease in pore size is in agreement with the reduction in fiber diameter for membranes with ZnO. For an enhanced wound healing a pore size sufficient to promote cell infiltration is required, according to Lowery, Datta and Rutledge (2010) investigation, pore sizes smaller than 6 μm don’t allow fibroblasts to penetrate the scaffold, while with pore sizes in the 20 μm as obtained in this work, the fibroblast cells are able to grow alongside single fibers, without forming bridges between fibers [52]. Although there is a slight variation in morphological traits among analyzed membranes, the properties are within the desired range for all the samples. These results are in accordance to the findings in Rodríguez‐Tobías et al. (2014) work, which analyzed ZnO addition effect up to 5 wt% in a PLLA electrospun membrane, with a slight reduction in fiber diameter and small variation in pore size and total porosity [53].

### 3.4 Wettability and Water Uptake

**FIGURE 4:**
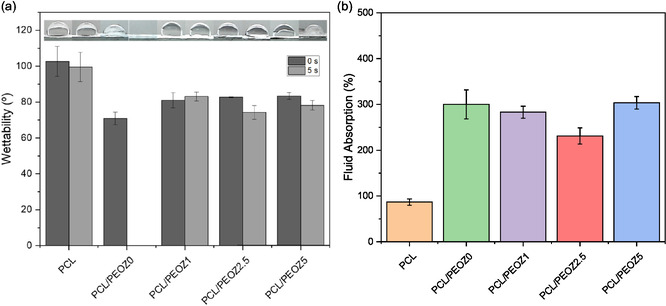
(a) Wettability measured by water contact angle assay and (b) fluid absorption for electrospun membranes with different ZnO concentrations. Error bars correspond to the standard deviation. Tests were conducted in triplicate.

Wettability was assessed to ensure the hydrophilic character of the tested material. The water contact angle was measured between a water droplet and material's surface in different times. The measured angles and image of the resulting droplet are shown in Figure 4a.

The pure PCL sample exhibits the highest contact angle among the analyzed samples, measuring 102.68° ± 8.31° at the moment of contact and 99.56° ± 8.11° after 5 s, indicating a low wettability and hydrophobic nature. In contrast, the PCL/PEOZ0 sample is the most hydrophilic, with an initial contact angle of 70.96° ± 3.52° and complete absorption of the water droplet within 5 s. The decrease in water contact angle is expected as the PEO's incorporation facilitates hydrogen bonding between PEO's oxygen molecule and water [54]. Similar water contact angles were reported for pure PCL membranes and membranes incorporating PEO into the polymeric matrix [21]. The incorporation of ZnO slightly reduces the wettability compared to PCL/PEOZ0; however, all ZnO‐containing samples maintain contact angles below 90°, indicating they remain hydrophilic. Prado‐Prone et al. [22] found a similar behavior in his work with ZnO loaded PCL/gelatin electrospun membranes, where an increase in ZnO content lead to a slight increase in the water contact angle, from 31.8° to 77.2°, with 0% ZnO and 6% ZnO content, respectively. However, in both cases, even with water contact angle increasing, it remains below the level of pure PCL. This effect may be associated with an interaction between PCL or PEO with ZnO in the form of intermolecular interactions of wan der Waals, which can reduce the surface energy of the fibers, thereby resulting in a increase in water contact angle.

Fluid Absorption assay is an important measure that indicates the capacity that the membrane has to absorb exudates in wound healing applications. Fluid absorption was used as a complementary analyze to wettability assay, and the results are presented in Figure 4b.

The PCL membrane exhibited the lowest fluid absorption capacity, retaining 86.63% ± 7.03% of its own mass, which is a similar result to other literature reports regarding pure PCL membranes, about 1 time its own mass after reaching equilibrium [55]. In contrast, samples containing 0, 1, 2.5, and 5 wt% ZnO exhibit significantly higher fluid absorption capacities, with values 300.12% ± 31.80%, 283.04% ± 13.07%, 231.13% ± 17.57%, and 303.53% ± 13.67%, respectively. The water uptake of the PCL/PEO membrane three times higher than that of pure PCL membrane is in accordance to Farzaei et al. [56] report, which found a 300% water uptake for a PCL/PEG membrane with 3:1 composition ratio after 24 h of immersion in water. We also found similar values in our previous work, which used chemically synthesized ZnO NPs loaded into the same PCL/PEO polymeric matrix [57]. The increased uptake with PEO addition is related to the presence of hydrophilic groups, swelling of the fibers and facilitated water entrance. These findings align with the wettability test results, indicating that the PCL/PEO composition significantly enhances hydrophilicity and water uptake capacity compared to pure PCL composition. Although the addition of ZnO led to a slight reduction in wettability, it did not appear to significantly impact the overall water uptake capacity, as the measurements were taken after the membranes had reached equilibrium with the liquid.

### 3.5 Cytotoxicity

**FIGURE 5:**
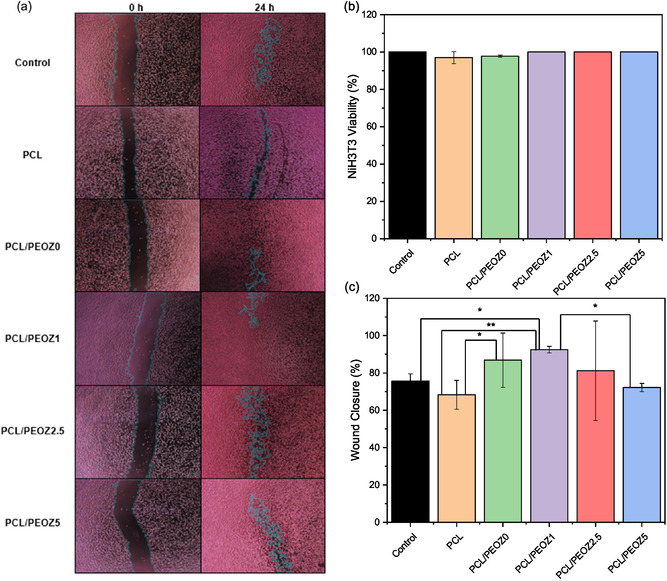
(a) Representative images of scratch test (cell migration) conducted with L929 cells cultured at 0 h and after 24 h of exposure to the tested membranes. (b) Cell viability of NiH3T3 cells and (c) wound closure rate of L929 cells in cell migration assay for the tested membranes. Error bars correspond to the standard deviation. Tests were conducted in quadruplicate. Statistical analysis performed considering *p* < 0.30 as statistically significant (* = *p* < 0.30, ** = *p* < 0.10).

Cytotoxicity tests are important for verifying the compatibility of the material with living cells. Cytotoxicity was assessed by MTT method, using a cellular lineage of murine fibroblasts (NiH3T3) cultured and incubated for 24 h with membrane samples with different ZnO NPs contents. The results were expressed by percentage of living cells and are shown in Figure 5b).

Figure 5b shows that all tested membranes exhibited high viability in NiH3T3 cells, with the ZnO‐containing membranes displaying remarkable results, reaching 100% cell viability. No statistically significant differences were observed among the experimental groups and the control, indicating the absence of cytotoxic effects on fibroblast cells for any of the evaluated formulations. The polymers utilized, namely PCL and PEO, have already been proven as noncytotoxic in many reports, and can even perform cell infiltration and proliferation enhancement and promote myofibroblast differentiation when processed as electrospun membranes that mimic the ECM [58, 59]. In addition, both ZnO nanoparticles and Moringa leaf extract isolated have been reported to stimulate cell proliferation and fibroblast differentiation, thus enhancing the desired healing effect [28, 29, 60]. These findings suggest that the membrane formulations with the green synthesized ZnO are not toxic for human cells, and have potential application as biomaterials.

### 3.6 In Vitro Cell Migration

Cell migration was assessed trough a scratch test using murine fibroblast L929 cells. Figure 5a shows representative images of L929 cells immediately after scratching (0 h) and after 24 h of exposure to each tested membrane. In each image, the area unfilled by cells is highlighted in blue. Tests were conducted in quadruplicate and analyzed using ImageJ software. Figure 5b presents the calculated percentage of wound closure for each membrane sample.

After 24 h, the pure PCL membrane achieved a wound closure of 68.28% ± 7.74%, compared to 75.65% ± 3.83% for the control, with no statistically significant difference. The membrane sample PCL/PEOZ0, composed of a PCL/PEO 7:1 blend without the addition of ZnO, exhibited 86.84% ± 14.56% of wound closure, higher than that of pure PCL membrane, with statistically significant different with *p* < 0.3 compared to the PCL membrane, but no statistical significance when compared to the control group. This improvement can be attributed to the increased wettability of PCL/PEOZ0 membrane due to the presence of PEO, which enhances cell affinity compared to the pure PCL. PCL/PEOZ1 membrane displayed a wound closure of 92.48% ± 1.78%, which represents the most significative difference from the control group with a *p* value of 0.2612 in relation to control, and a *p* value of 0.0533 in relation to the PCL group (one‐way ANOVA). With further increase in ZnO content the wound closure rate decreased to values closer to the control level, with 81.18% ± 26.62% and 72.16% ± 2.21% for PCL/PEOZ2.5 and PCL/PEOZ5, respectively. These results indicate that, at low concentrations, the synthesized ZnO may contribute to an increased ability of the membranes to support cell proliferation, a process associated with wound contraction and tissue regeneration. This behavior is consistent with literature reports on green‐synthesized ZnO NPs, which have demonstrated the capacity to stimulate fibroblast proliferation and to promote key events in the wound healing cascade, including inflammation modulation, enhanced collagen deposition, keratinocyte proliferation and re‐epithelialization, as well as increased neovascularization [49, 50]. However, the high variability observed in the assay limits the robustness of this interpretation, and additional in vitro studies, as well as in vivo wound closure experiments, are necessary to more accurately determine the material's potential to enhance wound healing. Moreover, although the scratch assay employs a fibroblast cell line as a simplified in vitro model, the wound healing process in vivo, particularly under infected conditions, involves complex enzymatic, inflammatory, and metabolic pathways, in which the behavior and biological effects of ZnO NPs may differ substantially and require further investigation [23, 61, 62, 63, 64].

### 3.7 Antimicrobial Activity

**FIGURE 6:**
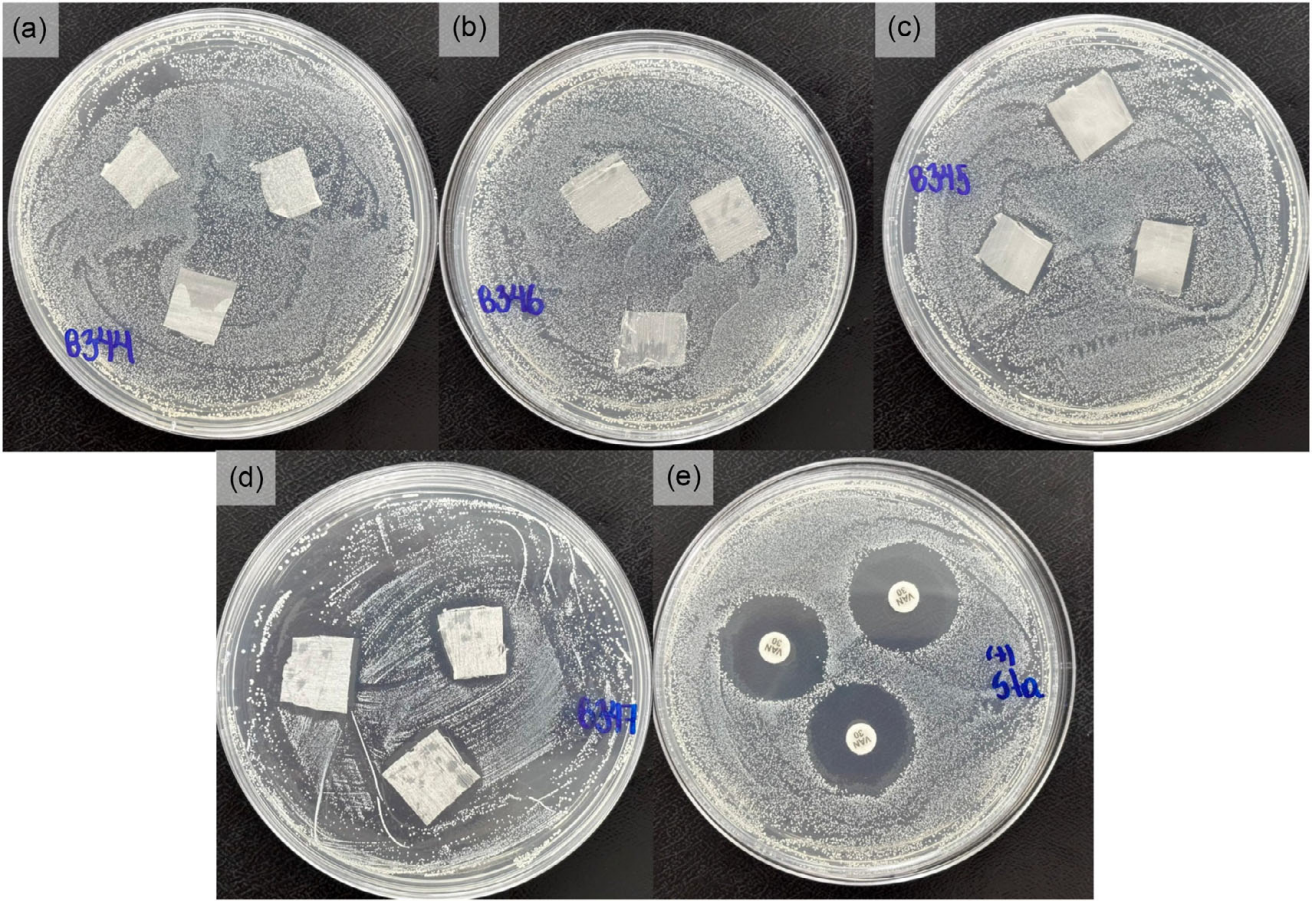
Inhibition halo formed by antimicrobial action from membranes of 10 × 10 mm against *Staphylococcus aureus*. Membranes with ZnO NPs addition in concentrations (a) 0%, (b) 1%, (c) 2.5%, (d) 5%, as well as (e) positive control.

**FIGURE 7:**
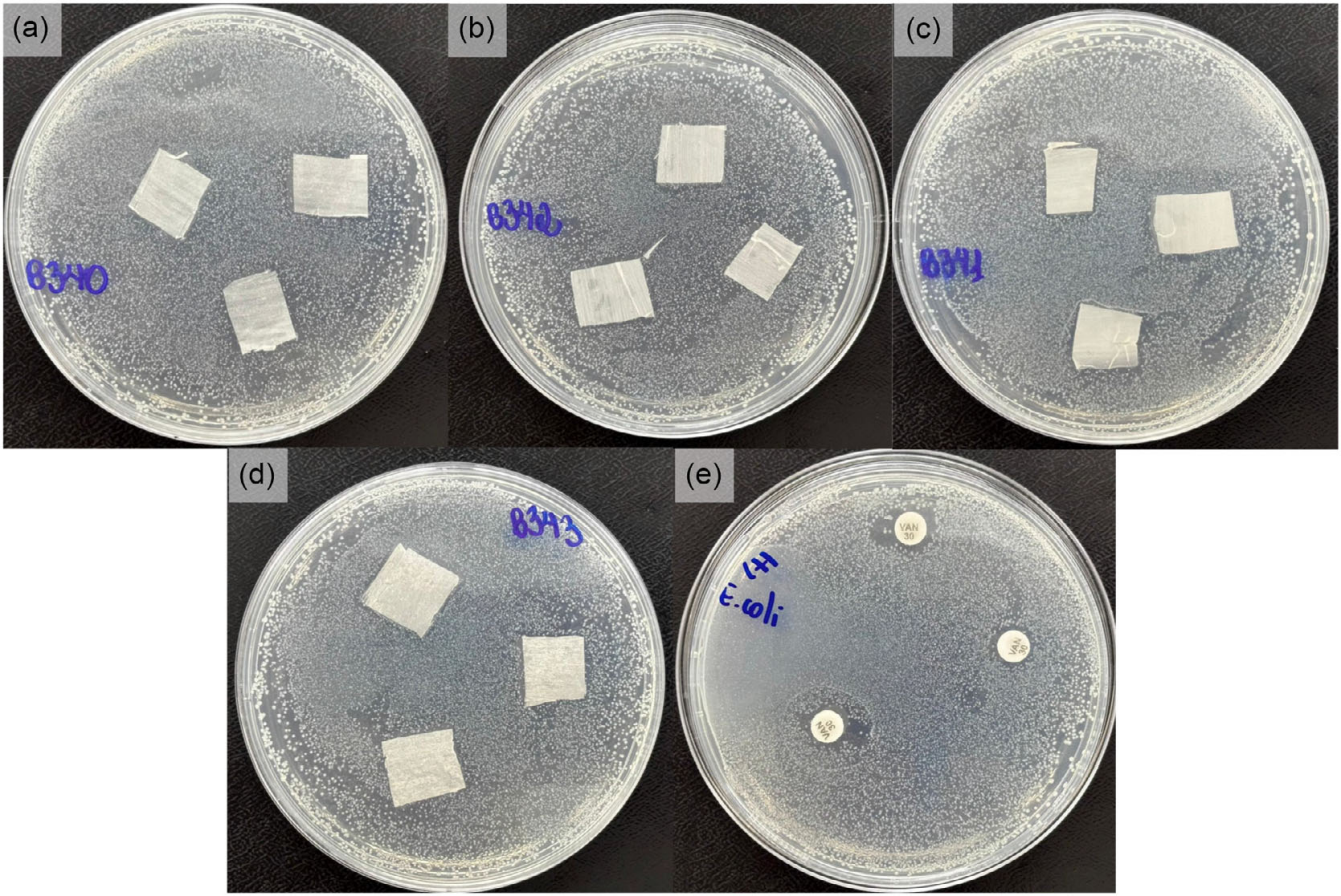
Inhibition halo formed by antimicrobial action from membranes of 10 × 10 mm against *Escherichia coli*. Membranes with ZnO NPs addition in concentrations (a) 0%, (b) 1%, (c) 2.5%, (d) 5%, as well as (e) positive control.

To determinate the potential of the ZnO loaded membranes in medical applications in the infected wound care, the antibacterial activity of the obtained material was assessed through the inhibition halo technique against *Staphylococcus aureus* and *Escherichia coli* bacteria, common Gram‐positive and Gram‐negative pathogens, respectively. Figure 6 depicts the resulting images of the antibacterial tests after 24 h of incubation for *Staphylococcus aureus*, while Figure 7 shows the results for *Escherichia coli*.

The inhibition zone values observed against both bacterial strains are summarized in Table 2. As expected, the PCL/PEO membrane without ZnO NPs did not exhibit any antibacterial activity, with no halo formation, due to the absence of an antimicrobial agent, and was therefore used as the negative control. For *Staphylococcus aureus* (Figure 6), membranes containing 2.5% and 5% ZnO NPs exhibited inhibition zones of approximately 1.0 and 2.0 mm, respectively, whereas the incorporation of 1% ZnO NPs was insufficient to produce detectable antibacterial activity. For *Escherichia coli* (Figure 7), all ZnO‐containing membranes displayed inhibition zone formation, however, the halo size observed for PCL/PEOZ5 membrane was smaller than that formed against S. aureus. This behavior can be attributed to structural differences between Gram‐positive and Gram‐negative bacteria. While Gram‐positive bacteria possess a relatively simple cell wall structure, Gram‐negative bacteria present an additional outer lipopolysaccharide membrane that acts as a protective barrier, reducing susceptibility to antimicrobial agents [65]. Overall, the inhibition zones observed in this study were smaller than those produced by the vancomycin control, his behavior may be associated with the limited diffusion of ZnO NPs from the polymeric membrane into the agar medium, which can restrict direct interaction between nanoparticles and bacterial cells, thereby reducing antibacterial efficacy [66]. Nevertheless, the inhibition zones obtained for the moringa‐derived ZnO NP‐loaded PCL/PEO membranes are consistent with previous reports on ZnO NP‐loaded electrospun systems. For instance, Augustine et al. [67] reported inhibition zone formation for ZnO‐containing PCL membranes at ZnO concentrations of 5% and 6%, while no antibacterial activity was observed for at lower concentrations in the range of 1 to 4%. In contrast, the present study demonstrated measurable inhibition zones at concentrations as low as 2.5% of ZnO for S. aureus and 1% for E. coli, which may be attributed to the synergistic effect of the PCL/PEO blend, as the hydrophilic nature of PEO promotes fiber swelling and partial dissolution upon contact with aqueous media, facilitating nanoparticle release into the surrounding environment when compared to pure PCL systems [21, 68].

**TABLE 2:** Inhibition zone formed by membrane's action.

Sample	Inhibition zone, mm	
	S. aureus	***E. coli***
PCL/PEOZ0	0.00 ± 0.00	0.00 ± 0.00
PCL/PEOZ1	0.00 ± 0.00	0.33 ± 0.577
PCL/PEOZ2.5	1.00 ± 0.00	1.00 ± 0.00
PCL/PEOZ5	2.00 ± 0.00	1.00 ± 0.00
Vancomycin 30 mg	8.0 ± 0.00	3.00 ± 0.00

Green‐synthesized nanoparticles have received significant attention for their antibacterial applications in recent years. ZnO NPs, in particular, exhibit strong antibacterial properties, primarily attributed to reactive oxygen species (ROS) production, which induces oxidative stress, protein and DNA damage, and cell membrane disruption, ultimately leading to bacterial cell death [50, 69]. The green synthesis approach enhances these effects by leveraging bioactive compounds in plant extracts, which not only act as reducing agents but also contribute additional antibacterial, antioxidant, and therapeutic properties [34, 35]. Among these, *Moringa oleifera* extract is particularly notable for its use in nanoparticle synthesis. Rich in polyphenols, flavonoids, carotenoids, alkaloids, and isothiocyanates, *Moringa oleifera* has demonstrated significant antibacterial, anticancer, and antioxidant potential [36, 37].

## 4 Conclusions

In this study, ZnO nanoparticles were synthesized through a green combustion method with *Moringa oleifera* extract and incorporated into PCL/PEO electrospun membrane for wound healing applications. The combustion synthesis successfully yielded ZnO with the characteristic wurtzite crystal structure. The moringa‐derived ZnO NPs exhibited a small crystallite size and a porous morphology, resulting from gas evolution during the combustion process, which contributed to an increased specific surface area, a desirable feature for biomedical applications. Notably, the adopted synthesis route enabled the incorporation of trace elements from *Moringa oleifera* leaves into the ZnO NPs, potentially transferring bioactive properties from the plant to the final material. The resulting composite membrane of PCL/PEO with ZnO addition exhibited a reduced water contact angle and an approximately threefold higher water uptake compared to pristine PCL membranes. This enhanced water affinity is particularly advantageous for wound care applications, as effective exudate management supports the maintenance of a moist wound environment conducive to tissue repair. Moreover, the increased fluid absorption capacity without rapid saturation may reduce the frequency of dressing changes, thereby improving patient comfort and minimizing disruption of newly formed tissue. The polymeric matrix of PCL and PEO was able to effectively delivers ZnO nanoparticles to the site, which is responsible for the significant antibacterial activity, demonstrated against *Staphylococcus aureus* and *Escherichia coli*. More important, the moringa‐ZnO nanoparticles exhibited selective cytotoxicity toward bacterial cells, without adversely affecting dermal cells, highlighting its suitability for wound healing applications, especially those where antibacterial properties are required. Overall, these findings suggest that the developed material is a promising functional wound dressing, capable of providing protection to the wound site, managing exudates, preventing and combating infections, and promoting accelerated tissue regeneration, making it particularly suitable for infected wounds. Further in vivo studies are required to assess the wound closure capacity and investigate potential anti‐inflammatory properties of the ZnO‐loaded membranes, which are essential steps toward future clinical translation and commercialization.

## Author Contributions

**Ronaldo Silveira**: conceptualization: (lead); investigation: (lead); methodology: (lead); visualization: (lead); writing – original draft: (lead). **Henrique Borba Modolon**: validation: (supporting); writing – review & editing: (supporting). **Edgar Andrés Chavarriaga Miranda**: conceptualization: (equal); investigation: (equal); methodology: (equal); validation: (equal); writing – review & editing: (equal). **Alex A. Lopera**: investigation: (supporting); methodology: (supporting); validation: (supporting). **Jaqueline Leite Vieira**: methodology: (supporting); validation: (supporting); writing review & editing: (supporting). **Milena Botelho Pereira Soares**: methodology: (supporting); validation: (supporting). **Josiane Dantas Viana Barbosa**: methodology: (supporting); supervision: (supporting). E**lidio Angioletto**: supervision: (supporting); validation: (supporting). **Tiago Bender Wermuth**: validation: (supporting); writing – review & editing: (supporting). **Oscar Rubem Klegues Montedo**: supervision: (supporting); validation: (supporting); writing – review & editing: (equal). **Sabrina Arcaro**: conceptualization: (equal); project administration: (lead); supervision: (lead); validation: (lead); visualization: (equal); writing – original draft: (lead).

## Funding

This study was supported by Conselho Nacional de Desenvolvimento Científico e Tecnológico (141985/2024‐0, 406510/2023‐7), Coordenação de Aperfeiçoamento de Pessoal de Nível Superior, and Fundação de Amparo à Pesquisa e Inovação do Estado de Santa Catarina (2024TR002563).

## Conflicts of Interest

The authors declare no conflicts of interest.

## Data Availability

Data sharing is not applicable to this article as no new data were created or analyzed in this study.
