# Correction to “Electrospun Poly(caprolactone)/Poly(ethylene oxide) Membranes Incorporating Green‐Synthesized Zinc Oxide Nanoparticles for Enhanced Wound Healing Applications”

**DOI:** 10.1002/smsc.70343

**Published:** 2026-07-22

1

Silveira, R., Modolon, H. B., Chavarriaga, E. A., Lopera, A. A., Vieira, J. L., Soares, M. B. P., Barbosa, J. D. V., Angioletto, E., Wermuth, T. B., Montedo, O. R. K., & Arcaro, S. (2026). Electrospun Poly(caprolactone)/Poly(ethylene oxide) Membranes Incorporating Green‐Synthesized Zinc Oxide Nanoparticles for Enhanced Wound Healing Applications. Small Science, 6(6), e202500562. https://doi.org/10.1002/SMSC.202500562.

The affiliation of the author Edgar Andrés Chavarriaga, currently listed as “Departamento de Fundamentación Básica, Institución Universitaria Pascual Bravo, Medellín, Colombia”. Is incorrect. This Should read “Departamento de ciencias ambientales y de la construcción, facultad de ciencias exactas y aplicadas, institución universitaria ITM, Medellín, Colombia.”

We apologize for this error.

